# Editorial: The Neuropsychiatry of Dreaming: Brain Mechanisms and Clinical Presentations

**DOI:** 10.3389/fneur.2021.666657

**Published:** 2021-03-25

**Authors:** Elemer Szabadi, Paul James Reading, Seithikurippu R. Pandi-Perumal

**Affiliations:** ^1^Developmental Psychiatry, Queen's Medical Centre, University of Nottingham, Nottingham, United Kingdom; ^2^South Tees Hospitals NHS Foundation Trust, Middlesbrough, United Kingdom; ^3^Somnogen Canada Inc., Toronto, ON, Canada

**Keywords:** dreaming, sleep paralysis, nightmares, sleep apnoea, agrypnia excitata, oneiric stupor, hypnotic dreams, mind wandering

Dreaming (involuntary mental activity during sleep, “sleep mentation”) has fascinated mankind for thousands of years (see e.g., Genesis 37, 40 in the Old Testament). Interestingly, Aristotle in the Fourth century BC seems to have recognized the similarity between hallucinations during waking and dreams during sleep: “…the faculty by which, in waking hours, we are subject to illusion when affected by disease, is identical with that produces illusory effects in sleep” (1). However, the medical relevance of dreaming has been recognized only for the last 100 years or so. The first step in this direction was made by Freud (2) who used patients' dreams to gain insight into their psychopathology. More recently, with development of insights into mechanisms underlying normal and abnormal sleep, a number of neuropsychiatric dream-related disorders have been recognized, such as rapid-eye movement-sleep- related behavior disorder (REM sleep behavior disorder, RBD) (3), sleep paralysis (4), hypnagogic/hypnopompic hallucinations (5), lucid dreaming (6), and nightmares (7). Although dreaming can occur both in rapid eye movement (REM) sleep and non-REM sleep, most of the neuropsychiatric disorders are associated with REM sleep.

There is a two-way relationship between brain science and the clinical neuropsychiatry of dream-related disorders: discoveries in the laboratory can help to unravel mechanisms underlying clinical disorders, and reports of clinical symptoms and syndromes (“nature's experiments”) can give impetus to laboratory research. The aim of the Research Project was to collect papers both from the field of dream-related clinical neuropsychiatric disorders and from laboratory-based dream research that focuses on the underlying mechanisms of these disorders, and thus promote insights into their functional neuropathology.

As dreams are subjective experiences they are not easily available for research. One simple metric uses dream recall frequency. Putois et al. reviewed the factors that may influence dream recall in laboratory dream research. These include demographic variables (gender and age), sleep variables (sleep stages, chronotype, duration of intra-sleep awakenings, time elapsed between awakening and dream report, and the sleeper's environment), psychological variables (previous waking experiences, memory, and personality), methods for studying dreams, sleep disorders, psychopathology, and substances. It is emphasized that it is essential, when comparing different groups of dreamers, that any observed difference is due to experimental variables and not uncontrolled variables between dreamers. Nicolas and Ruby reviewed the effects of psychotropic drugs on dreaming (dream recall frequency and content). Antidepressants and sedative psychotropic drugs reduce dream recall frequency, mostly by improving sleep quality and reducing intra-sleep awakenings. The effect of psychotropic drugs on dream recall frequency does not seem to be related to their effect on REM sleep. Although there is some understanding of the brain mechanisms mediating the effects of psychotropic drugs on sleep (8), little is known of the ways by which these drugs may modify dreaming.

A number of objective neural correlates of dreaming have been established (9) that have revealed evidence of dreaming in the absence of dream recall (10). Scarpelli et al. examined the EEG correlates of dream recall in elderly subjects. It was found that frontal theta oscillations during the last segment of REM sleep before awakenings were predictive of dream recall after awakening. This finding confirmed previously reported observations in healthy young subjects.

Not surprisingly, sleep disorders are associated with changes in dreaming. BaHammam and Almeneessier reviewed dreaming in obstructive sleep apnoea (OSA), a common sleep disorder characterized by sleep fragmentation, oxygen desaturation and excessive daytime sleepiness. OSA has contradictory effects on dream recall: while most studies report a decrease in dream recall frequency that increases on continuous positive airway pressure (CPAP) treatment, there are also reports of increased dream recall frequency in OSA with increased emotional (hostile, violent) content. Future studies should aim at establishing whether there is any difference in dreaming in REM sleep-predominant and non-sleep-stage-specific dreaming.

Sleep paralysis is a REM sleep parasomnia due to intrusion of REM sleep atonia into wakefulness, sometimes with dream mentation with visual or tactile perceptions. It is associated with intense fear and perception of threat. Sleep paralysis is a symptom of narcolepsy, but it can also occur in the absence of narcolepsy (“isolated sleep paralysis”). Jalal et al. examined the effectiveness of Mediation-Relaxation (MR) therapy, a psychological treatment, in a pilot study involving 10 patients presenting with narcolepsy-associated sleep paralysis. MR therapy was compared with a control intervention (deep breathing). MR therapy resulted in a dramatic decrease in the number of days when sleep paralysis occurred. Future work should explore MR therapy in a larger cohort of narcoleptic patients and extend it to patients with isolated sleep paralysis.

As reviewed by Putois et al., psychopathology is one of the factors that can influence dreaming. Psychopathology can be associated not only with a change in dream recall frequency, but also with changes in the quality and contents of dreams. Earlier work in adults has demonstrated that “dysphoric dreaming” (bad dreams and nightmares) are associated with daytime (waking) psychopathology (11). Gauchat et al. extended this work to children by examining the relationship between disturbing dreams and psychosocial maladjustment. They found that disturbed dreaming was associated with both internalizing and externalizing behaviors. Furthermore, most externalizing behaviors were moderated by early negative emotionality, illustrating the importance of temperamental traits.

Although dreaming by definition occurs in sleep, there are reports of dream-like experiences in the absence of sleep in some states of altered consciousness. Two such conditions are considered in the Research Topic: agrypnia excitata and hypnosis. Agrypnia excitata is a rare severe sleep/wakefulness disorder, characterized by persistent insomnia and motor and autonomic activation. It can be associated with “oneiric stupor,” an unusual disorder of dreaming. Oneiric stupor is characterized by the recurrence of stereotyped gestures mimicking daily-life activities and reporting dream mentation of a single dream-like scene. Baldelli and Provini reviewed the phenomenology of oneiric stupor together with its possible physiological basis.

It has been shown that explicit suggestions to have a dream in a hypnotic state can evoke a dream-like experience (“hypnotic dreaming”). Fazekas and Nemeth discussed the nature of hypnotic dreams, and concluded that they are more akin to mind-wandering than dreaming.

## Author Contributions

All authors listed have made a substantial, direct and intellectual contribution to the work, and approved it for publication.

## Conflict of Interest

SP-P was employed by Somnogen Canada Inc. The remaining authors declare that the research was conducted in the absence of any commercial or financial relationships that could be construed as a potential conflict of interest.
